# Effects of Exhaustive Aerobic Exercise on Tryptophan-Kynurenine Metabolism in Trained Athletes

**DOI:** 10.1371/journal.pone.0153617

**Published:** 2016-04-28

**Authors:** Barbara Strasser, Daniela Geiger, Markus Schauer, Hannes Gatterer, Martin Burtscher, Dietmar Fuchs

**Affiliations:** 1 Medical University Innsbruck, Division of Medical Biochemistry, Biocenter, Innsbruck, Austria; 2 Medical University Innsbruck, Division of Biological Chemistry, Biocenter, Innsbruck, Austria; 3 Department of Sport Science, Medical Section, University of Innsbruck, Innsbruck, Austria; Macquarie University, AUSTRALIA

## Abstract

Exhaustive exercise can cause a transient depression of immune function. Data indicate significant effects of immune activation cascades on the biochemistry of monoamines and amino acids such as tryptophan. Tryptophan can be metabolized through different pathways, a major route being the kynurenine pathway, which is often systemically up-regulated when the immune response is activated. The present study was undertaken to examine the effect of exhaustive aerobic exercise on biomarkers of immune activation and tryptophan metabolism in trained athletes. After a standardized breakfast 2 h prior to exercise, 33 trained athletes (17 women, 16 men) performed an incremental cycle ergometer exercise test at 60 rpm until exhaustion. After a 20 min rest phase, the participants performed a 20 min maximal time-trial on a cycle ergometer (RBM Cyclus 2, Germany). During the test, cyclists were strongly encouraged to choose a maximal pedalling rate that could be maintained for the respective test duration. Serum concentrations of amino acids tryptophan, kynurenine, phenylalanine, and tyrosine were determined by HPLC and immune system biomarker neopterin by ELISA at rest and immediately post exercise. Intense exercise was associated with a strong increase in neopterin concentrations (p<0.001), indicating increased immune activation following intense exercise. Exhaustive exercise significantly reduced tryptophan concentrations by 12% (p<0.001) and increased kynurenine levels by 6% (p = 0.022). Also phenylalanine to tyrosine ratios were lower after exercise as compared with baseline (p<0.001). The kynurenine to tryptophan ratio correlated with neopterin (r = 0.560, p<0.01). Thus, increased tryptophan catabolism by indoleamine 2,3-dioxygenase appears likely. Peak oxygen uptake correlated with baseline tryptophan and kynurenine concentrations (r = 0.562 and r = 0.511, respectively, both p<0.01). Findings demonstrate that exhaustive aerobic exercise is associated with increased immune activation and alterations in monoamine metabolism in trained athletes which may play a role in the regulation of mood and cognitive processes.

## Introduction

Exhaustive exercise has been associated with a transient depression of immune function [1]. Already moderate physical activity significantly impacts on inflammation cascades that involve several pro-inflammatory cytokines like interferon-y (IFN-y) and down-stream biochemical pathways [2, 3]. Recently, two studies reported detailed insights into the physiological mechanisms involved under extreme conditions [4, 5]. One study noted a significant increase of the reactive oxygen species (ROS) production rate and oxidative damage after a mountain ultra-marathon running [4]. This includes also changes in the concentrations of urinary neopterin, which is predominantly produced by human monocytes/macrophages, and elevation of which is often linked with conditions of immune activation and inflammation [6]. A second study revealed changes in serum free amino acids during a half-ironman triathlon [5].

Further, data indicates significant effects of immune activation cascades on the biochemistry of monoamines such as essential amino acid tryptophan (TRP), the precursor molecule of 5-hydroxy-tryptamine (5HT, serotonin) [7]. Serotonin plays a key role in signal transduction between neurons, and exercise-induced changes in serotonin concentrations have been linked to central fatigue [8]. However, TRP is not only precursor of the serotonin biochemical pathway which is conducted by enzyme tryptophan 5-hydroxylase but is also the key element for the formation of nicotinamide-adenine-dinucleotides NAD and NADH via the so-called kynurenine (KYN) pathway [9]. The latter is either achieved by hepatic tryptophan 2,3-dioxygenase (TDO) or by indoleamine 2,3-dioxygenases 1 and 2 (IDO1 and IDO2). TDO is controlled by TRP levels and can be also induced by steroid hormones like cortisol [10]. However, pro-inflammatory stimuli like Th1-type cytokine IFN-y strongly induce IDO1 expression and activity during cellular immune response, and accelerated TRP breakdown is indicated by an increased KYN to TRP ratio (KYN/TRP) [11].

IFN-y is the most important stimulus of indoleamine 2,3-dioxygenase-1 (IDO1) activity although other mainly pro-inflammatory stimuli can do the same [7]. In long distance runners, significantly increased production of IFN-y and also other relevant cytokines was reported in urine [2]. In this early study, IFN-y remained undetectable in serum, most probably because of lacking sensitivities of available assays. However, the parallel increase of KYN/TRP and neopterin concentrations in our study further supports the involvement of IFN-y. Moreover, exercise increased IDO1 activity of macrophages was reported in rats [12]. Still until now there is no direct information available on the effect of exercise on IFN-y and IDO1 activity in humans as any effect of other pro- and anti-inflammatory cytokines by exercise on IDO1 activity remains to be examined.

It should not be neglected that in addition, stimulation of TDO can increase KYN/TRP. However, a contribution of IDO1 rather than TDO to the enhanced TRP breakdown can be substantiated when an association of KYN/TRP with a biomarker of immune activation, such as neopterin, can be demonstrated [7], because in parallel to IDO1, IFN-y induces also GTP-cyclohydrolase 1 (GCH1), the key enzyme for the production of neopterin [6].

Enhanced serum KYN/TRP levels are frequent in patients suffering from diseases which go along with immune activation and inflammation such as infections, autoimmune syndromes or malignant tumors [7]. Thereby, IDO1 catalyses the formation of KYN and, hence, limiting availability of TRP for serotonin biosynthesis in the brain. Indeed, the probability of depression and fatigue development is increased in such clinical conditions [13, 14]. Because of its immunotolerizing properties [15], the upregulation of IDO1 activity may also represent a key component of exercise-induced immunosuppression in athletes, e.g., immune system disturbances following intense exercise have been linked to an increased risk to upper-respiratory tract infections among athletes [16].

Another important aspect in the pathogenesis of depression and fatigue might represent the phenylalanine–tyrosine pathway which is controlled by enzyme phenylalanine 4-hydroxylase (PAH) and represents the starting point of the biosynthetic pathway of noradrenergic, adrenergic and dopaminergic neurotransmitters. The activity of the enzyme is reflected by the phenylalanine (PHE) to tyrosine (TYR) ratio (PHE/TYR). Recently pro-inflammatory cascades were found to be associated with a disturbed PAH activity [17, 18]. Moreover, in older adults with chronic low-grade inflammation, not only disturbed TRP metabolism but also PHE and TYR metabolism was associated with neuropsychiatric symptoms [19].

The aim of the present study was to investigate the effect of exhaustive aerobic exercise on biomarkers of immune activation and metabolism of the amino acids TRP, KYN, PHE, and TYR in trained athletes. We hypothesized that exhausting exercise is associated with an accelerated TRP breakdown, shifts of PHE/TYR metabolism, and increased neopterin production, indicating increased immune activation following intense exercise.

## Materials and Methods

### Ethics Statement

All the participants were informed of the risks and discomforts associated with the investigation and signed a written consent to participate. The study was approved by the Board for Ethical Questions in Science Ethics of the University Innsbruck according to the principles expressed in the Declaration of Helsinki.

### Subjects

Thirty-three healthy and trained athletes volunteered to participate in this study. Participants were excluded if they had a previous history of muscle disorder, cardiac or kidney disease or those taking medicine (including anti-inflammatory drugs, antibiotics, supplements), nicotine, or consuming regularly alcohol (>10/20 g for women and men, respectively, per day). A questionnaire about their medical history and previous training was filled out by each participant. Selected baseline characteristics of the 33 eligible individuals (17 women, 16 men) in this investigation are presented in Table 1.

**Table 1:** Baseline characteristics, aerobic fitness for the participants in this study.

Variable	mean ± SD
n (males/females)	16/17
Age (yrs)	26.7 ± 3.7
BMI (kg/m^2^)	21.7 ± 2.0
Height (cm)	173 ± 8.1
Weight (kg)	65.5 ± 10.6
Endurance training (h/wk)	7.3 ± 3.5
Peak power output (W/kg)	4.6 ± 0.5
Time-trial mean power output (WTT)	205 ± 47

Endurance training refers to training of the aerobic system, mainly continuous endurance training at moderate intensity (60% to 80% peak oxygen uptake), covered per week in the month prior to the test.

### Experimental Protocol

In the morning of the exercise test a standardized breakfast was provided 2 hours prior to exercise (379 kcal; 88 energy percent carbohydrates, 11 energy percent proteins, and 1 energy percent fat). For eligibility testing all subjects performed an incremental cycle ergometer exercise test until exhaustion. The cycle ergometry was performed on an electronically braked ergometer (Ergometrics 900, Ergoline, Germany) and started at a workload of 50/75 W (women/men) for 5 minutes (warm up) with a following increase in workload of 25 W per minute until exhaustion. Exhaustion was assumed when the pedalling rate became below 60 rpm. Heart rate and ventilatory parameters were monitored continuously (Oxycon mobile, Jaeger, Germany). Peak power output (W_max_) was defined as the last completed workload rate plus the fraction of time spent in the final uncompleted work rate multiplied by 25 W [20]. Peak oxygen uptake (VO_2max_) was defined as the highest 30-second average during the test.

After a 20 minutes resting period, athletes with a maximal aerobic capacity of ≥150% of reference values for cardiorespiratory response [21] performed a 20-minute maximal time-trial on a cycle ergometer (RBM Cyclus 2, Germany) as described by Faulhaber and colleagues [20]. Briefly, the cycle ergometer was shifted to a fixed pedal force in which power output was dependent on the pedalling rate. Pedal force for each participant was set so that pedalling at 100 rpm would produce about 70% (rounded to 5 W) of peak power output, which was determined by the incremental cycle ergometry. During the test, cyclists were strongly encouraged to choose a maximal pedalling rate that could be maintained for the respective test duration. The main outcome measurement was mean power output during the 20-min test, which was automatically calculated by the software of the ergometer. The participants were allowed to drink water ad libitum.

### Blood measurements

We conducted blood collections in supine position from a medial cubital vein before exercise and within 5 min post exercise. After centrifugation for 10 minutes plasma was removed and samples were frozen at −20°C until analysis. Neopterin concentrations were measured by ELISA (BRAHMS Diagnostics, Hennigsdorf, Germany) following the manufacturer’s instructions. Serum concentrations of free TRP and KYN as well as concentrations of phenylalanine (PHE) and tyrosine (TYR) were determined by high-performance liquid chromatography (HPLC), as previously described [22, 23]. The ratios of KYN/TRP and PHE/TYR were calculated as indexes of TRP breakdown and PAH activity, respectively.

### Statistical Analysis

Statistical analyses were conducted by SPSS (IBM SPSS Statistics Version 22). Normality in the distribution of data was tested by the Kolmogorov-Smirnov's test. In dependency of Gaussian distribution, the Student’s t test for paired samples or the Mann-Whitney-U-test was carried out to assess significant differences in changes of the same variables before and after the intervention (results of parametric and non-parametric calculations were very similar). Spearman’s rank correlation was used to assess the association between two variables. A p-value of less than 0.05 (two-tailed) was considered to indicate statistical significance. Data are presented by mean values ± standard deviation (SD) for baseline characteristics, for biomarkers of immune activation and metabolism of amino acids by mean values ± standard error of the mean (SEM).

## Results

Females had a lower body mass index (BMI), VO_2max_, and mean power output during the 20-min test (W_TT_) compared to male athletes, as KYN levels were lower in females (U = 2.342, p = 0.019). None of the other parameters was influenced by gender. Concentrations of biomarkers of immune activation and metabolism of amino acids following intense exercise are listed in Table 2.

**Table 2:** Biological markers before (pre) and after (post) an exhaustive aerobic exercise in 33 athletes.

Variable	Pre mean ± SEM	Post mean ± SEM	P-value
Neopterin (nmol/L)	6.4 ± 0.56	10.2 ± 0.97	< 0.001
Tryptophan (μmol/L)	65.1 ± 1.87	57.1 ± 1.65	< 0.001
Kynurenine (μmol/L)	1.88 ± 0.08	1.99 ± 0.09	0.022
KYN/TRP (μmol/mmol)	29.0 ± 1.07	35.1 ± 1.43	< 0.001
Tyrosine (μmol/L)	134 ± 4.66	141 ± 3.90	0.018
Phenylalanine (μmol/L)	69.3 ± 1.59	68.7 ± 1.28	n.s.
PHE/TYR (μmol/μmol)	0.53 ± 0.01	0.49 ± 0.01	< 0.001

KYN/TRP kynurenine to tryptophan ratio

PHE/TYR phenylalanine to tyrosine ratio

n.s. not significant

At baseline, neopterin concentrations correlated with KYN/TRP levels (r = 0.608, p<0.0001), a biomarker of TRP catabolism. However, there was only a moderate association between neopterin and TRP and KYN concentrations (r = -0.297 and r = 0.306, respectively, p>0.05). No such correlations existed between neopterin and PHE or TYR metabolism.

Exhausting exercise was associated with a strong increase in neopterin levels (by 159% of baseline, p<0.001) and this increase was significantly influenced by endurance training volume with a strong negative correlation between athletes' training status and concentrations of neopterin at exhaustion (r = -0.502, p = 0.006).

**Fig 1:**
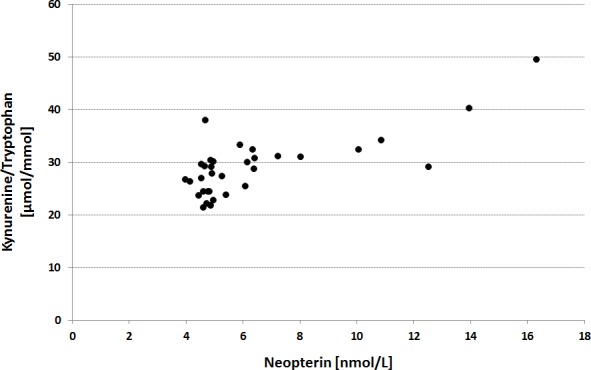
Association between neopterin and kynurenine to tryptophan concentrations (KYN/TRP) in 33 trained athletes after exhaustive aerobic exercise. The correlation of neopterin with post-exercise KYN/TRP ratio, a biomarker of tryptophan catabolism, was statistically significant (r = 0.560, p = 0.001).

In parallel, TRP breakdown was significantly induced by exercise as indicated by a decline in TRP levels by 12% (p<0.001) and an increase of KYN levels by 6% (p<0.02), accompanied by an elevation of KYN/TRP by 20% (p<0.001). Concentrations of neopterin still correlated with KYN/TRP levels (r = 0.560, p = 0.001; Fig 1), Further, both TYR levels and PHE/TYR significantly increased with intense exercise (p = 0.018 and p<0.001, respectively). There was no significant change of PHE concentrations induced by exercise (Table 2).

VO_2max_ correlated significantly with baseline concentrations of TRP (r = 0.562, p = 0.001) and KYN (r = 0.511, p = 0.002) and this relation remained significant for KYN after exercise (r = 0.359, p = 0.04). W_TT_ correlated with KYN levels before (r = 0.534, p = 0.001) and after exercise (r = 0.409, p = 0.018) but not with baseline and post-exercise TRP concentrations. No significant associations existed between PHE-TYR metabolism and all sport physiological variables (S1 and S2 Files).

## Discussion

Results of this study show that exhaustive aerobic exercise in well trained athletes elicits significant biochemical alterations which are related to inflammation and immune activation cascades. They may also shed some new light on the shift of TRP levels between different body compartments which has been documented recently by Areces et al. [5]. The decline of essential amino acid TRP in our study was much stronger as compared with the observations after the half iron-marathon where the alteration of absolute TRP levels failed to reach statistical significance, and only the decline of the ratio of TRP *vs*. branch chained amino acids (BCAA) was significant [5]. Still the drastic decline of TRP in our study could relate to muscle fatigue experienced after intense physical exercise.

Our results indicate an involvement of IDO1 activation in the enhanced TRP catabolism and KYN production following intense exercise, because a strong association was found between neopterin and KYN/TRP levels throughout the study suggestive of IDO1-induced TRP catabolism. This observation could be of special relevance for several pathophysiological consequences that are induced by physical exercise. The decline of TRP by IDO1 is well established as an immunoregulatory event [24] which is related to the development of regulatory T-cells and dampening immune response as a kind of feedback control [15].

In the present study, the resulting low TRP levels followed by intense exercise may diminish brain supply, thereby reducing TRP availability to the brain for serotonin production. The decline of TRP was drastic and would certainly also diminish the TRP to BCAA ratio that is most relevant for the transport of TRP into the brain via the leucine-preferring L1 transporter system. Alternatively, down-stream catabolites of kynurenine like quinolinic acid and picolinic acid are formed which could be of physiological importance especially regarding their possible interference with neuroendocrine circuits like the interaction with the N-methyl-D-aspartate (NMDA) receptor [25–28]. Thus, the stimulated TRP breakdown could relate to mood alterations related to physical exercise and may contribute to a declining training adherence in athletes.

**Fig 2:**
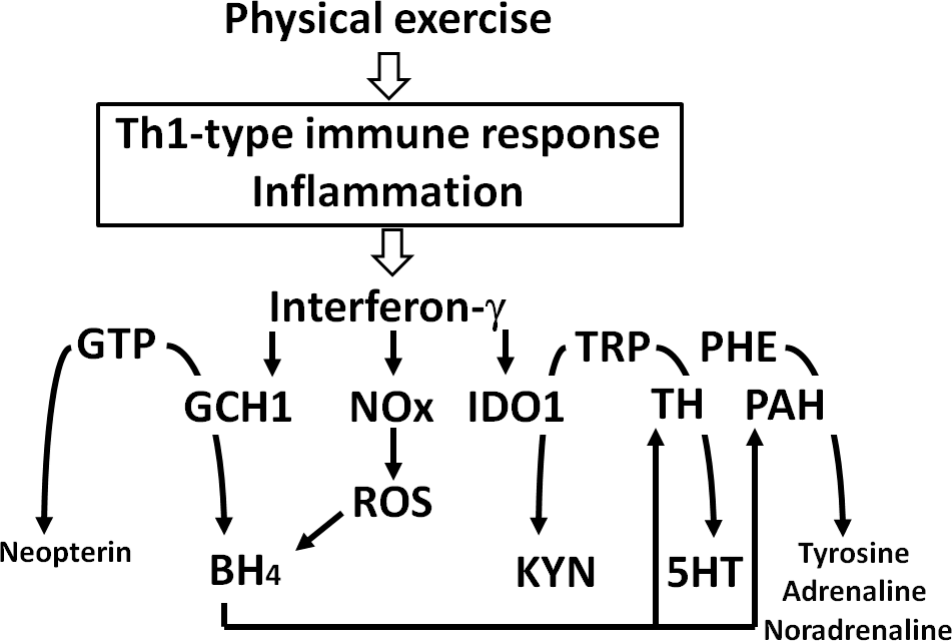
**Divergent effects of moderate vs. exhaustive physical exercise on the production of neurotransmitters:** Pro-inflammatory cytokines like interferon-y (IFN-y) stimulates several enzymes including [a] indoleamine 2,3-dioxygenase-1 (IDO1), which degrades tryptophan (TRP) and serotonin (5HT), [b] NADPH oxidase (NOx), which produces reactive oxygen species (ROS), and [c] GTP-cyclohydrolase-1 (GCH1), which in human macrophages forms neopterin and in other cells tetrahydrobiopterin (BH_4_), the necessary cofactor of several amino acid hydroxylases, including tryptophan 5-hydroxylase (TH) for the production of 5-hydroxytryptamine (5HT, serotonin) and phenylalanine 4-hydroxylase (PAH) for the production of tyrosine, precursor of dopamine, adrenaline and noradrenaline. Physical exercise is followed by a pro-inflammatory immune response which induces BH_4_, thereby upregulating several neurotransmitters (see pathways marked in red), associated with mood enhancement and well-being. However, when physical exercise or training is too heavy and exhaustive, tryptophan breakdown by IDO1 becomes too drastic and the decline of tryptophan due to IDO1 activity can no longer be compensated by BH_4_, the life span of the latter is decreased by ROS exposure. Thus, athletes may suffer from insufficient supply with neurotransmitters and will experience low mood.

In our study, baseline TRP metabolism was found to be associated with aerobic fitness. Higher levels of both TRP and KYN at baseline were associated with a higher aerobic capacity (VO_2max_). On the other hand, athletes with higher training volumes in the month prior to the test demonstrated a smaller rise in neopterin concentrations to intense exercise, suggesting lower immune activation with better training status. In addition, we found a significant decline of PHE/TYR following exercise, suggesting a higher activity of PAH induced by exercise. Such an increase may indicate a stimulated production of the noradrenergic, adrenergic and dopaminergic neurotransmitters, which could contribute to an improved neuropsychiatric presentation [18]. Thus, the higher production rate of the neurotransmitters may compensate, at least partly, the mood lowering effect of intense exercise [29], potentially caused by the decline of TRP. The increase of PAH activity, indicated by PHE/TYR, could result from the increased activity of GCH1, which is reflected by the increase of monocyte-macrophage-derived neopterin that is usually accompanied by a similar increase of 5,6,7,8-tetrahydrobiopterin (BH_4_), the cofactor of PAH and also tyrosine hydroxylase, as well as tryptophan hydroxylase, enzymes that are all important in the formation of relevant neurotransmitters [30] (Fig 2).

With this respect it is important to note that moderate *vs*. intense physical activity might have contrasting effects. On the one hand, physical exercise may enhance the production of neurotransmitters by induction of BH_4_ biosynthesis and increasing neurotransmitter biosynthesis which will contribute to mood enhancement (Fig 2). However, the pro-inflammatory cascades that are initiated are accompanied by production of ROS, which may diminish the endogenous antioxidant pools, including BH_4_ concentrations [31]. Breakdown of TRP by IDO1 will follow and slows down serotonin formation when TRP becomes diminished. Consequently, BH_4_-dependent biosynthesis of several neurotransmitters will decline and the mood enhancement after moderate exercise will be followed by a decline when sports become too heavy or training intervals too intense. It could play a role in the onset of fatigue and sleep disturbances [29, 32].

One may conclude that sports performed occasionally with two or three days interval exerts a beneficial effect on general well-being as it is the case when it is performed as recreational activity whereas intense training will more and more achieve adverse effects on both mood [29] and immune system [33]. Prolonged bouts of strenuous exercise have been shown to result in transient depression of white blood cell functions and it is suggested that such changes create an “open window” of decreased host protection, increasing the risk of developing an infection.

The current investigation presents some **limitations** with respect to the experimental design and the measurements obtained. Our findings are certainly limited by the relatively small sample size, so that we were not able to analyze specific subgroups. This would be especially important when effects of gender could be of relevance, e.g., females presented with lower KYN levels and VO_2max_ compared to males. Still, peak power output (W/kg) was not influenced by gender and, further, the effect of exercise on KYN/TRP or neopterin concentrations was seen in both subgroups and, thus, independent of gender. Future studies should consider gender effects in a larger population since an influence of sex steroid hormones like estrogen on TDO is well established [10]. In a similar way, a potential influence of cortisol on TDO activity cannot be ruled out [9]. Salivary and serum cortisol levels in humans and corticosterone in rats are increased by exercise [34, 35] and this could induce liver TDO, as demonstrated in rats. The combined effects of increased liver TRP and TDO induction can elevate serum KYN in humans [36] and rats [12]. In both of these latter studies, increases in serum KYN after exercise have been shown. Thus, also an additional role of TDO in the exercise-induced TRP breakdown cannot be fully ruled out.

Further, we did not measure NAD^+^ levels and downstream NAD^+^ related pathways in the present study. Future work should address the regulatory influence of NAD^+^ on the expression of sirtuins (SIRT) and PGC-1α because enhanced SIRT1 and PGC-1α activity is associated with improved mitochondrial function and exercise performance [37], and protection against obesogenic feeding [38].

## Conclusions

In summary, our study demonstrates a significant influence of exhaustive aerobic exercise on biochemical pathways which are linked to inflammation and immune activation responses induced by exercise. Intense exercise was associated with a strong increase in neopterin levels. In parallel, TRP breakdown was significantly induced by exercise, accompanied by an elevation of the KYN/TRP ratio, suggestive of IDO1-induced TRP catabolism. The stimulated TRP breakdown could relate to mood alterations related to physical exercise and may play a role in the onset of fatigue.

## Supporting Information

S1 File (PDF)

S2 File (PDF)
