# Supplementary material for: Effects of Exhaustive Aerobic Exercise on Tryptophan-Kynurenine Metabolism in Trained Athletes
Source: PLoS One. 2016 Apr 28;11(4):e0153617. doi: 10.1371/journal.pone.0153617 (PMC4849644; doi:10.1371/journal.pone.0153617)
Supplement: S1 File — (PDF) [file pone.0153617.s001.pdf]

Strasser

12.03.2015

	Allergosan			1=m/2=fm	
Rot	**Pr.Nr.**		**Age**	**Sex**	**BMI**
1	CS-1-B-1	B	24	2	20,6
2	FM-2-B-1	B	32	1	21,9
3	SN-3-B-1	B	21	1	22,4
4	SS-4-B-1	B	24	2	24,3
5	MG-5-B-1	B	31	1	24,4
6	DL-6-B-1	B	22	1	23,3
7	FS-7-B-1	B	22	1	25,5
8	CB-8-B-1	B	25	2	21,2
9	TW-9-B-1	B	26	1	21,3
10	PB-10-B-1	B	34	1	23,3
11	CF-11-B-1	B	28	2	18,6
12	JS-12-B-1	B	26	1	23,4
13	CLS-13-B-1	B	27	2	22,9
14	MS-14-B-1	B	28	1	25,3
15	PW-15-B-1	B	25	1	22,7
16	AG-16-B-1	B	28	2	22,6
17	LW-17-B-1	B	27	2	22,1
18	AR-18-B-1	B	23	1	23,2
19	TB-19-B-1	B	25	2	21,8
20	IB-20-B-1	B	25	2	17,0
21	FF-21-B-1	B	32	1	25,4
22	VM-22-B-1	B	28	2	19,9
23	TR-23-B-1	B	24	2	20,9
24	MM-24-B-1	B	31	2	21,7
25	MAS-25-B-1	B	20	2	19,1
26	IF-26-B-1	B	31	1	22,5
27	SM-27-B-1	B	31	2	19,6
28	CK-28-B-1	B	24	1	21,6
29	KK-29-B-1	B	30	2	19,6
30	BH-30-B-1	B	25	1	22,1
31	MH-31-B-1	B	25	2	20,7
32	SK-32-B-1	B	23	2	19,3
33	RL-33-B-1	B	35	1	24,4
Blau					
1	CS-1-E-1	E	24	2	20,6
2	FM-2-E-1	E	32	1	21,9
3	SN-3-E-1	E	21	1	22,4
4	SS-4-E-1	E	24	2	24,3
5	MG-5-E-1	E	31	1	24,4
6	DL-6-E-1	E	22	1	23,3

7	FS-7-E-1	E	22	1	25,5
8	CB-8- E-1	E	25	2	21,2
9	TW-9-E-1	E	26	1	21,3
10	PB-10-E-1	E	34	1	23,3
11	CF-11-E-1	E	28	2	18,6
12	JS-12-E-1	E	26	1	23,4
13	CLS-13-E-1	E	27	2	22,9
14	MS-14-E-1	E	28	1	25,3
15	PW-15-E-1	E	25	1	22,7
16	AG-16-E-1	E	28	2	22,6
17	LW-17-E-1	E	27	2	22,1
18	AR-18-E-1	E	23	1	23,2
19	TB-19-E-1	E	25	2	21,8
20	IB-20-E-1	E	25	2	17,0
21	FF-21-E-1	E	32	1	25,4
22	VM-22-E-1	E	28	2	19,9
23	TR-23-E-1	E	24	2	20,9
24	MM-24-E-1	E	31	2	21,7
25	MAS-25-E-1	E	20	2	19,1
26	IF-26-E-1	E	31	1	22,5
27	SM-27-E-1	E	31	2	19,6
28	CK-28-E-1	E	24	1	21,6
29	KK-29-E-1	E	30	2	19,6
30	BH-30-E-1	E	25	1	22,1
31	MH-31-E-1	E	25	2	20,7
32	SK-32-E-1	E	23	2	19,3
33	RL-33-E-1	E	35	1	24,4

ml/kg/min	W/kg	h/wk	h/wk	Watt	mmol/l	nmol/L
**VO2max**	**Wmax**	**Training (pre)**	**WNTZ (post)**	**TT**	**Lac**	**Neopterin**
36,9	3,60	6	2,92			4,7
40,2	3,80	9	6,12			4,7
66,2	5,10	10	9,27			4,5
56,1	4,90	15	7,10			4,6
53,7	4,60					14,0
57,8	4,60	10	9,42			4,9
48,2	4,00	7	4,62			4,8
52,8	4,70	6	6,13			4,6
64,0	5,40	15	3,97			6,4
58,0	4,70	15	13,40			4,9
50,2	4,60	6	8,57			4,9
59,4	4,80	15	8,88			4,7
48,3	4,20	8	6,45			5,9
50,9	4,30	10	9,15			4,0
60,7	4,80	10	9,37			4,9
47,4	4,50	15	5,67			4,8
45,9	4,40	10	5,30			6,4
50,3	5,30	8	5,45			6,1
39,9	3,70					10,9
39,2	3,80	6	3,93			12,5
52,0	4,60					6,2
49,3	5,20	8	8,37			7,2
45,6	4,80	10	5,77			10,1
57,4	4,90	30	21,00			5,3
45,5	4,20	6	4,30			4,6
50,3	4,30	20	5,40			16,3
48,1	4,70	20	10,83			4,4
63,3	5,20	14	6,30			4,1
44,3	4,50	6	5,33			6,4
59,1	5,20	15	6,57			5,0
41,0	4,00	15	6,67			5,4
49,1	4,40	6	5,28			5,0
64,1	5,20					8,0

36,9				141	9,6	14,0
40,2				175	10,33	4,9
66,2				248	11,08	8,5
56,1				213	9,49	5,3
53,7				273	12,24	21,4
57,8				257	11,35	7,6

48,2				216	7,05	4,8
52,8				169	9,73	7,1
64,0				251	11,71	16,7
58,0				248	8,01	4,9
50,2				173	11,43	6,8
59,4				256	14,4	11,8
48,3				176	6,8	9,0
50,9				255	11,61	4,4
60,7				273	10,01	4,7
47,4				166	14,27	4,8
45,9				169	10,72	16,4
50,3				247	14,4	8,1
39,9				139	3,63	13,3
39,2				117	8,12	22,5
52,0				237	6,72	9,3
49,3				177	14,01	14,5
45,6				194	10,53	13,7
57,4				185	10,25	7,3
45,5				139	12,01	9,2
50,3				200	9,29	20,5
48,1				170	10,48	4,9
63,3				229	10,69	4,6
44,3				162	11,42	11,4
59,1				274	13,51	7,2
41,0				173	12,7	9,1
49,1				164	10,11	10,5
64,1				290	11,94	16,6
	4,58	11	7,29			
	0,4780201	5,46788266	3,45			

µmol/L	µmol/L	µmol/mmol	µmol/l	µmol/l	mol/mol
**Trp**	**Kyn**	**Kyn/Trp**	**Tyr**	**Phe**	**Phe/Tyr**
58,4	1,29	22,1	137,2	65,9	0,48
64,8	1,90	29,3	143,8	69,3	0,48
70,1	2,08	29,6	128,8	67,3	0,52
62,1	1,67	26,9	142,2	57,3	0,40
53,0	2,14	40,3	127,6	63,1	0,49
99,0	3,00	30,3	116,0	70,9	0,61
49,5	1,21	24,4	130,7	63,2	0,48
71,9	1,54	21,4	155,1	68,0	0,44
71,9	2,33	32,4	138,2	65,4	0,47
67,8	1,98	29,1	118,8	66,1	0,56
52,7	1,47	27,9	113,0	61,5	0,54
66,8	2,54	38,0	175,8	70,1	0,40
50,5	1,68	33,3	109,8	66,9	0,61
60,1	1,61	26,7	116,6	76,0	0,65
87,7	1,91	21,7	202,5	84,9	0,42
57,5	1,41	24,4	146,7	67,7	0,46
59,1	1,70	28,7	153,7	71,1	0,46
64,0	1,63	25,4	161,4	82,1	0,51
51,1	1,74	34,1	94,3	64,6	0,69
61,5	1,79	29,1	89,7	57,5	0,64
65,6	1,97	30,0	160,7	80,9	0,50
65,2	2,03	31,1	151,0	75,5	0,50
63,2	2,05	32,4	152,0	74,4	0,49
68,2	1,86	27,3	159,5	92,1	0,58
68,2	1,66	24,4	130,7	86,1	0,66
61,3	3,04	49,5	107,4	60,4	0,56
65,7	1,55	23,6	151,1	74,5	0,49
75,4	1,98	26,3	124,1	70,9	0,57
65,6	2,01	30,7	105,9	67,9	0,64
73,9	2,22	30,1	117,9	59,4	0,50
59,2	1,41	23,7	140,7	63,1	0,45
73,7	1,68	22,8	87,0	57,2	0,66
63,4	1,96	31,0	129,4	65,1	0,50

50,9	1,41	27,7	140,9	57,9	0,41
57,8	2,20	38,0	121,2	58,6	0,48
51,2	2,10	41,0	159,5	68,5	0,43
59,7	1,92	32,1	135,5	57,6	0,42
46,1	2,61	56,7	137,9	67,1	0,49
54,5	1,73	31,8	134,3	66,1	0,49

61,9	1,87	30,2	155,0	67,9	0,44
53,9	1,52	28,2	164,4	74,6	0,45
59,0	2,25	38,1	173,8	75,4	0,43
61,5	2,10	34,2	132,8	72,1	0,54
57,2	1,46	25,6	142,7	70,9	0,50
55,0	2,57	46,7	171,2	75,2	0,44
42,2	1,54	36,6	120,1	60,1	0,50
51,2	1,54	30,2	103,1	60,0	0,58
83,2	2,11	25,4	201,3	87,3	0,43
64,5	1,97	30,6	138,1	59,5	0,43
47,5	1,65	34,7	158,3	72,8	0,46
57,8	1,92	33,1	145,0	66,0	0,46
51,5	1,44	28,0	123,2	73,4	0,60
85,9	3,54	41,2	114,0	65,4	0,57
45,7	1,45	31,7	155,9	68,9	0,44
57,0	2,24	39,3	136,8	65,6	0,48
54,4	2,16	39,7	167,0	77,3	0,46
61,6	1,94	31,5	132,8	76,0	0,57
59,0	1,90	32,3	126,7	77,1	0,61
51,3	3,00	58,6	124,4	71,3	0,57
60,1	1,70	28,3	156,0	76,2	0,49
59,5	2,06	34,6	137,7	69,6	0,51
56,4	1,96	34,7	113,5	63,0	0,55
60,8	2,20	36,1	136,3	63,4	0,47
49,0	1,51	30,8	143,4	73,3	0,51
61,9	1,80	29,1	106,0	61,6	0,58
54,7	2,30	42,0	139,1	68,0	0,49
