# Supplementary material for: Effects of Exhaustive Aerobic Exercise on Tryptophan-Kynurenine Metabolism in Trained Athletes
Source: PLoS One. 2016 Apr 28;11(4):e0153617. doi: 10.1371/journal.pone.0153617 (PMC4849644; doi:10.1371/journal.pone.0153617)
Supplement: S2 File — (PDF) [file pone.0153617.s002.pdf]

Descriptive Statistics

visit 1:	N	Mean	SD	SEM	Mean	SD	SEM
ages	33	26,73	3,82	0,71			
sex	33	1,52	0,51	0,09			
bmi	33	21,96	2,05	0,38			
Vo2	33	51,37	7,86	1,45	post	post	
neo	33	6,40	3,00	0,56	10,2	5,25	0,97
trp	33	65,1	10,1	1,87	57,1	8,90	1,65
kyn	33	1,88	0,42	0,08	1,99	0,47	0,09
kyntrp	33	29,0	5,77	1,07	35,1	7,70	1,43
phe	33	134	25,2	4,66	141	21,1	3,90
tyr	33	69,3	8,59	1,59	68,7	6,92	1,28
phetyr	33	0,53	0,08	0,01	0,49	0,06	0,01
watt	33	205	47,7	8,84			
lac	33	10,6	2,42	0,45			

Spearman's rho (visit 1)		sex	bmi	VO2	neo0	trp0	kyn0
age	rs	-0,106	0,174	0,057	0,294	-0,192	0,169
	p(2-tailed)	0,558	0,333	0,751	0,097	0,285	0,346
	n	33	33	33	33	33	33
sex	rs		-,666**	-,643**	0,073	-0,299	-,503**
	p(2-tailed)		0,000	0,000	0,685	0,091	0,003
	n		33	33	33	33	33
bmi	rs			,480**	-0,045	-0,142	0,172
	p(2-tailed)			0,005	0,804	0,431	0,337
	n			33	33	33	33
VO2	rs				-0,180	,562**	,511**
	p(2-tailed)				0,317	0,001	0,002
	n				33	33	33
neo0	rs					-0,297	,381*
	p(2-tailed)					0,093	0,028
	n					33	33
trp0	rs						,463**
	p(2-tailed)						0,007
	n						33
kyn0	rs						
	p(2-tailed)						
	n						
kyntrp0	rs						
	p(2-tailed)						
	n						
tyr0	rs						
	p(2-tailed)						
	n						
phe0	rs						

	p(2-tailed)
	n
phetyr0	rs
	p(2-tailed)
	n
watt	rs
	p(2-tailed)
	n
lac	rs
	p(2-tailed)
	n
neo1	rs
	p(2-tailed)
	n
trp1	rs
	p(2-tailed)
	n
kyn1	rs
	p(2-tailed)
	n
kyntrp1	rs
	p(2-tailed)
	n
tyr1	rs
	p(2-tailed)
	n
phe1	rs
	p(2-tailed)
	n

MWU test

U	p
4,75	<0,001
3,73	<0,001
2,29	0,022
2,66	<0,001
2,36	0,018
0,19	n.s.
3,66	<0,001

kyntrp0	tyr0	phe0	phetyr0	watt	lac	neo1	trp1
0,339	0,063	0,054	-0,051	0,129	-0,029	0,089	-0,131
0,054	0,729	0,767	0,778	0,473	0,874	0,621	0,466
33	33	33	33	33	33	33	33
-0,268	-0,054	-0,067	0,080	-,828**	-0,127	0,162	0,019
0,132	0,765	0,712	0,659	0,000	0,480	0,366	0,916
33	33	33	33	33	33	33	33
0,243	0,197	0,066	-0,234	,706**	-0,061	-0,218	-0,212
0,173	0,272	0,714	0,189	0,000	0,737	0,224	0,236
33	33	33	33	33	33	33	33
0,170	0,165	0,076	-0,149	,828**	0,202	-0,191	0,096
0,345	0,359	0,675	0,406	0,000	0,260	0,287	0,597
33	33	33	33	33	33	33	33
,608**	-0,193	-0,247	0,163	-0,023	-0,048	,762**	-0,209
0,000	0,282	0,166	0,366	0,898	0,791	0,000	0,244
33	33	33	33	33	33	33	33
-0,163	0,175	0,318	0,049	0,318	0,153	-0,197	,353*
0,365	0,330	0,071	0,784	0,071	0,394	0,273	0,044
33	33	33	33	33	33	33	33
,750**	-0,106	0,017	0,114	,534**	0,147	,391*	-0,114
0,000	0,558	0,925	0,526	0,001	0,415	0,024	0,526
33	33	33	33	33	33	33	33
	-0,267	-0,167	0,194	0,315	0,067	,565**	-,392*
	0,134	0,353	0,280	0,074	0,712	0,001	0,024
	33	33	33	33	33	33	33
		,619**	-,709**	0,193	0,226	-0,167	0,022
		0,000	0,000	0,283	0,206	0,353	0,904
		33	33	33	33	33	33
			0,010	0,088	0,184	-0,266	-0,058

0,958	0,625	0,305	0,135	0,748
33	33	33	33	33
	-0,226	-0,184	0,093	0,006
	0,206	0,306	0,606	0,972
	33	33	33	33
		0,276	-0,154	-0,054
		0,120	0,393	0,765
		33	33	33
			0,062	0,007
			0,732	0,967
			33	33
				-,392*
				0,024
				33

kyn1	kyntrp1	tyr1	phe1	phetyr1
0,207	0,210	-0,143	-0,004	0,168
0,248	0,240	0,426	0,984	0,351
33	33	33	33	33
-,414*	-0,331	-0,175	0,000	0,195
0,017	0,060	0,330	1,000	0,277
33	33	33	33	33
0,090	0,158	0,122	-0,177	-0,318
0,617	0,379	0,498	0,323	0,071
33	33	33	33	33
,359*	0,229	0,332	0,201	-0,262
0,040	0,199	0,059	0,263	0,141
33	33	33	33	33
0,324	,441*	-0,100	0,087	0,211
0,065	0,010	0,580	0,632	0,239
33	33	33	33	33
0,244	0,023	0,145	0,293	-0,038
0,172	0,899	0,421	0,098	0,832
33	33	33	33	33
,664**	,705**	0,020	0,176	0,019
0,000	0,000	0,913	0,328	0,918
33	33	33	33	33
,472**	,738**	-0,141	0,007	0,129
0,006	0,000	0,435	0,969	0,473
33	33	33	33	33
0,004	-0,095	,710**	,379*	-,609**
0,982	0,601	0,000	0,030	0,000
33	33	33	33	33
-0,130	-0,133	0,240	,373*	-0,054

0,471	0,460	0,178	0,032	0,765
33	33	33	33	33
-0,114	0,014	-,732**	-0,123	,804**
0,529	0,940	0,000	0,494	0,000
33	33	33	33	33
,409*	0,326	0,310	0,073	-0,299
0,018	0,064	0,080	0,686	0,091
33	33	33	33	33
0,301	0,266	0,205	-0,005	-0,092
0,088	0,134	0,252	0,980	0,610
33	33	33	33	33
0,306	,560**	0,000	0,081	0,096
0,083	0,001	1,000	0,654	0,596
33	33	33	33	33
0,331	-0,199	-0,085	0,030	0,021
0,060	0,266	0,637	0,869	0,909
33	33	33	33	33
	,782**	0,016	0,043	-0,053
	0,000	0,929	0,814	0,771
	33	33	33	33
		-0,054	-0,057	0,018
		0,765	0,751	0,92
		33	33	33
			,540**	-,730**
			0,001	0,000
			33	33
				0,091
				0,616
				33
